# Intestinal T-cell lymphomas NOS presenting as a polypoidal lesion: A case report

**DOI:** 10.1097/MD.0000000000038465

**Published:** 2024-06-07

**Authors:** Hanxin Bi, Junfang Bai, Limei Wang, Cong Liang, Ying Wu

**Affiliations:** a The Second People’s Hospital of Shaanxi Province, Xi’an, Shaanxi Province, China.

**Keywords:** colonoscopy, intestinal T-cell lymphomas NOS, polypoidal lesion

## Abstract

**Rationale:**

Intestinal T-cell lymphomas are exceedingly rare diseases. Intestinal T-cell lymphoma NOS, as a “wastebasket” category, is difficult to diagnosis. Endoscopy can identify abnormal mucosa in most patients at a reasonably early stage. Therefore, it is crucial to increase the understanding of endoscopists in terms of the endoscopic characteristics of ITCL.

**Patient concerns:**

A 74‐year‐old male alone with wasting as the major complaint, had multiple polypoid lesions in the large intestine. The patient then had endoscopic care.

**Diagnoses:**

Only 1 polypoid lesion on white-light endoscopy in the sigmoid colon was pathologically diagnosed as intestinal T-cell lymphomas, not otherwise specified (ITCL-NOS).

**Interventions:**

The patient underwent intensity-reduced CHOP therapy.

**Outcomes:**

The patient is still with controlled disease but developed chemotherapy-related side effects.

**Lessons:**

In the individual with unexplained anemia and waste, endoscopy should not be delayed. For each of polypoid lesion on white-light endoscopy, the endoscopist need to remain cautious, because every lesion in the same patient can exhibit the independence of histopathological features. Meanwhile, we suggest that endoscopists should routinely observe the terminal ileum, even take biopsy samples if necessary.

## 1. Introduction

The gastrointestinal tract (GI) is the most frequent extra-nodal site of non-Hodgkin lymphomas, accounting for 30% to 40% of all cases.^[1]^ Colorectal lymphomas with a lower rate of involvement than either stomach or small intestine involvement, overwhelmingly belong to the B-cell lineage, and T-cell origin is rarer. Primary intestinal T-cell lymphomas represent 10% to 16% of GI lymphomas and are exceedingly rare and aggressive extra-nodal lymphoid malignancies.^[2]^ 2022 5th edition of the World Health Organization (WHO) classification, and the 2022 international consensus classification (ICC) has reorganized entities that were listed as mature T- and NK-cell neoplasms in the 4th WHO classification, intestinal T-cell and NK-cell lymphoid proliferations and lymphomas are classified into 5 subtypes: Enteropathy-associated T-cell lymphoma (EATL), Monomorphic epitheliotropic ITCL (MEITL), Indolent T-cell lymphoma of the gastrointestinal tract, Indolent NK-cell lymphoproliferative disorder of the gastrointestinal tract, and ITCL not otherwise specified (NOS).^[3,4]^

The diagnosis of ITCL is challenging due to its uncommon clinical and histological symptoms and low incidence. Pathological diagnosis by biopsy is the most reliable diagnostic modality. Endoscopy, which can identify abnormal mucosa in most patients at a reasonably early stage, is a helpful adjunct. Here we report a 74‐year‐old man, only with wasting as a chief complaint, who found multiple polypoid lesions in the large intestine during a colonoscopy. Only 1 polypoid lesion in the sigmoid colon was pathologically classified as intestinal T-cell lymphomas, not otherwise specified (ITCL-NOS) by biopsy.

## 2. Case report

### 2.1. Investigations

In June 2023, a 74-year-old male with coronary heart disease and hypertension was hospitalized at our hospital for wasting. The patient reported losing 10 kg of weight and experiencing an appetite reduction over the past 3 years. Laboratory testing showed a decreased hemoglobin level of 92 g/L (normal value: 130–175 g/L), a low albumin level of 30.1g/L (normal value: 40–55 g/L), a positive result on a fecal occult blood test, and a negative result in oncological series. In the meantime, computed tomography (CT) scans of the abdomen and chest revealed no anomalies. Upon gastroscopy, no unusual findings were discovered. The patient underwent a colonoscopy with macroscopic findings of polyps or polypoid lesions. Subsequently, this patient underwent 2 rounds of endoscopic therapy because of the enormous volume and numerous polyps.

### 2.2. Diagnosis

**Figure 1.**
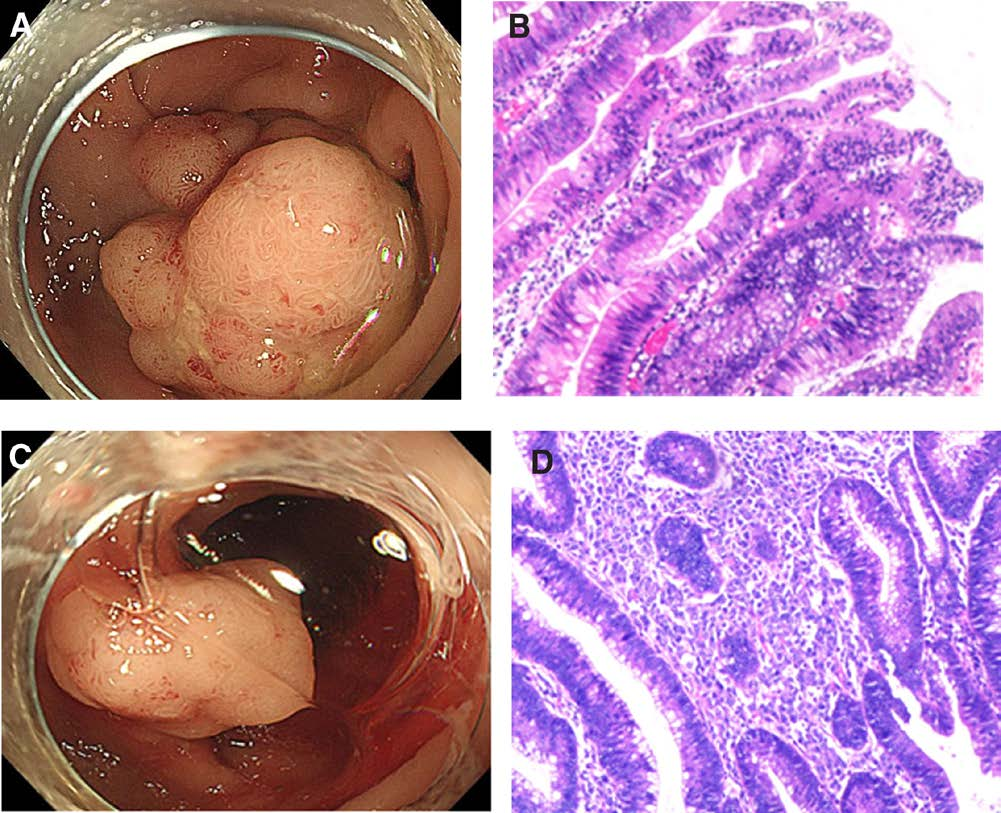
Imaging of endoscopic manifestation and corresponding histological features. (A) Colonoscopy images showing a polypoid lesion in the adjacent sigmoid colon diagnosed as tubular adenomas with partial high-grade intraepithelial neoplasia (sizes for 3.0 cm × 1.5 cm × 1.5 cm). (B) (H&E stain, ×100) Histopathological findings of this polypoid lesions. (C) Colonoscopy images showing a polypoid lesion in the adjacent sigmoid colon diagnosed as ITCL-NOS (sizes for 1.5 cm × 1.2 cm × 0.8 cm). (D) (H&E stain, ×100) Histopathological findings of this polypoid lesions. ITCL-NOS = intestinal T-cell lymphomas, not otherwise specified.

**Figure 2.**
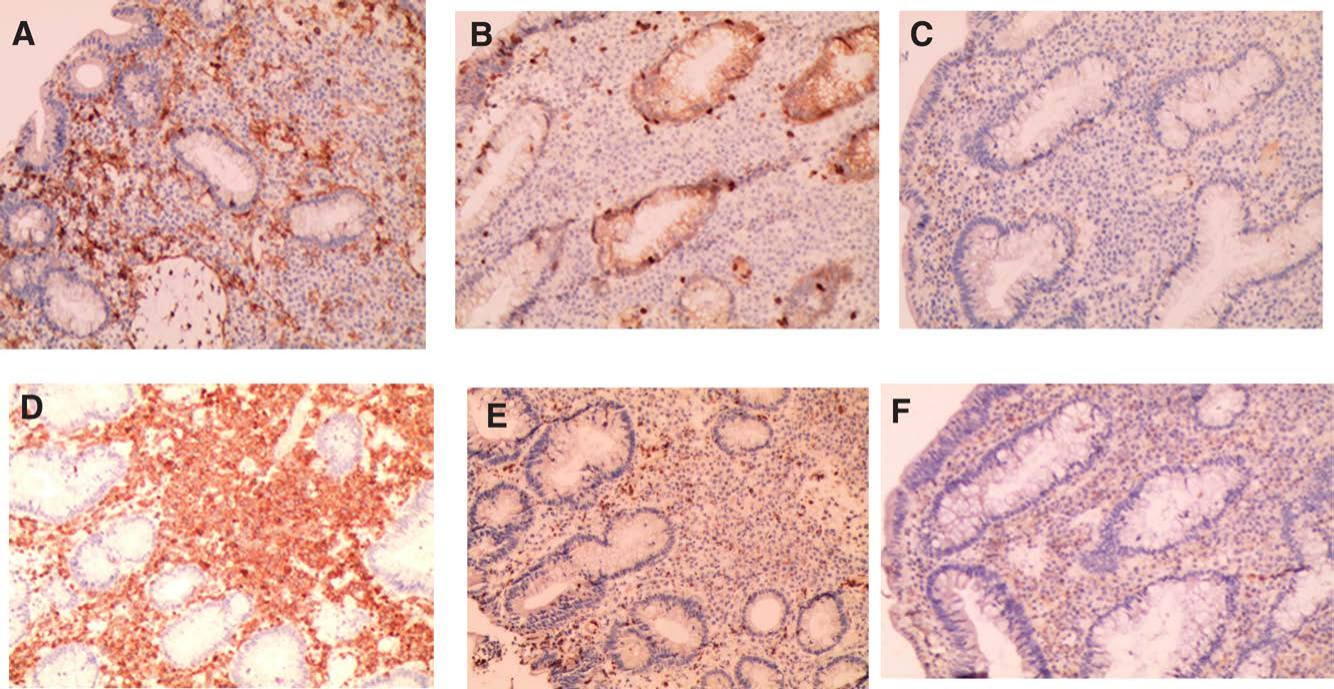
Immunohistochemical characterization of the atypical lymphocytic infiltrates. Infiltrated lymphocytes were negative for CD4 (A), CD8 (B), and CD56 (C), but positive for CD3 (D), TIA-1 (E), CD30(weak positive, (F), ×100.

**Figure 3.**
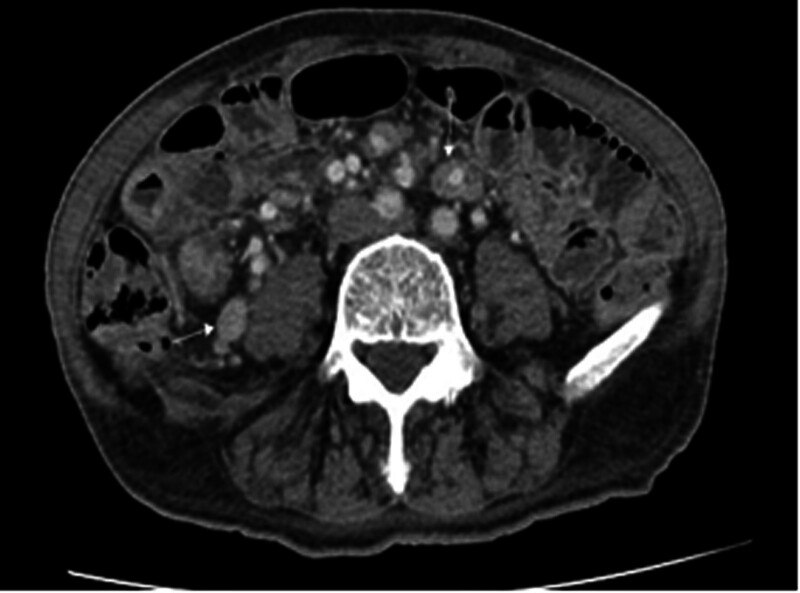
Contrast-enhanced CT images: Enlarged lymph nodes scattered in the retroperitoneum, and around the branch of the superior mesenteric artery. CT = computed tomography.

More than 10 pedunculated or nonpedunculated 10- to 20-mm polypoid lesions were exposed during endoscopy, part of the polyps has a villous look. Endoscopic mucosal resection (EMR) is used for larger polyps（＜20 mm）. Cold snare polypectomy is used for diminutive polyps（＜10 mm）. The great majority of polypoid lesions are classified as adenoma by histology, with partial high-grade intraepithelial neoplasia. Only 1 polypoid lesion of the sigmoid (sizes for 1.5 cm × 1.2 cm × 0.8 cm) revealed abnormal lymphocytes with obvious nuclear atypia presented as a lamellar or nodular distribution under the microscope, and the intestinal wall was destroyed so that poorly differentiated malignant tumors were prioritized (Fig. 1). Immunohistochemical stains demonstrated that the lymphoid infiltrates were positive for CD3, GranzymeB, Vim, TIA-1, CD30 (weak positive), and were negative for CD4, CD8, CD20, CD56, HMB-45, S100, MelanA, ALK, EMA, as well as negative EBV-encoded small RNA. About 80% of the Ki-67 labeling index was high (Fig. 2). The diagnosis of ITCL-NOS resulted from these observations. Expanded lymph nodes were seen in the retroperitoneum and surrounding the superior mesenteric artery branch on a later contrast-enhanced CT scan of the chest and abdomen (Fig. 3). Positron emission tomography-CT (PET-CT) showed the accumulation of fluorodeoxyglucose in multiple lymph nodes including the colon, small bowel, gastric fundus, corpus, and even neck, and mediastinum, these match the features of lymphoma. At last, he was sent to the hematology department.

### 2.3. Follow-up and outcomes

The patient received 8 cycles of intensity reduction of the CHOP regimen, we noticed an increase in his appetite and body weight, and the patient condition was under control. Yet, he experienced a compression fracture of the vertebra during treatment. After several months of conservative treatment, the patient local symptoms were relieved. Subsequently, we performed colonoscopy reexamination for the patient in March 2024 (in the 9 months later), only found 3 polypoid lesions about 0.3 cm in the descending and sigmoid colon. A subsequent biopsy later revealed chronic inflammatory and lymphocytic infiltration, also the high concentration of lymphoid tissue in the terminal ileum was found by biopsy. We will continue to follow-up and observe this patient.

## 3. Discussion

The clinical, histopathological, and molecular features of the ITCL differ among the various entities. According to the latest WHO 2022 classification of tumors of hematopoietic and lymphoid tissues, intestinal T-cell lymphomas NOS, a diagnosis of exclusion, is a trash basket category used for all the primary ITCL lacking the diagnostic criteria for other ITCL subtypes. We ruled out the potential of EATL since our patient lacked conclusive clinical, serological, or histological data supporting a diagnosis of celiac disease.^[5,6]^ The most prevalent form of ITCL in Asia is called MEITL, and it often involves a tumor infiltration that is CD8 + and CD56 + and exhibits a pronounced epitheliotropic pattern and monomorphic look. Since the patient did not exhibit these particular symptoms, this diagnosis was disregarded.^[7,8]^ Superficial cytologic atypia and the elevated proliferation index preclude the diagnosis of indolent intestinal T-cell lymphoma which has a proliferation index of more than 10% on Ki-67.^[9,10]^ EBV status matters, the EBER in situ hybridization was negative for abnormal lymphocytes in this case. The likelihood of NK-cell enteropathy was also ruled out by the lack of CD56.^[11]^ As a result, we believe that ITCL-NOS could be a plausible diagnosis for our patient.

As the endoscopic technique evolves, gastrointestinal lymphomas diagnosed with endoscopy are increasing. Meanwhile, the initial complaints of these patients were typically related to gastrointestinal symptoms, including abdominal pain, nausea, vomiting, altered bowel habits, and even intestinal obstruction and perforation.^[12]^ Gastrointestinal endoscopy can identify most abnormal mucosa in a relatively early stage. However, relevant studies about ITCL are rare due to low incidence and lack of endoscopic findings specificity. The few existing studies have reported the most common endoscopic abnormalities in intestinal lymphoma are the ulcerated lesion followed by eminence lesion, in addition to the diffusely infiltratively lesion and lymphomatous polyposis lesion et al.^[12–14]^ Ulcerated lesions may present multiple or solitary, various in size, different in shades, irregular in shape, the edges are flat or irregularly thickened. Yet those performance is often misdiagnosed as inflammatory bowel disease.^[15]^ Eminence lesions frequently appear as single or multiple polypoid or nodular lesions with or without mucosal ulceration at the top. We report the patient found multiple polypoid lesions in the large intestine. Only 1 polypoid lesion in the sigmoid colon treated with EMR was finally diagnosed as ITCL-NOS. To our knowledge, these special endoscopic findings for ITCL-NOS have not been reported previously. Curiously, there was no significant difference between this lesion and several other adenoma polyps on white-light endoscopy. Moreover, the lesion had no obvious top ulceration, even smaller than another polypoid lesion diagnosed as tubulovillous adenomas with high-grade intraepithelial neoplasia in the adjacent sigmoid colon (sizes for 3.0 cm × 1.5 cm × 1.5 cm).

Also, the study found that the small intestine is the most common location for intestinal lymphoma, as well the ileocecum is commonly reported.^[14]^ In our case, the patient small intestinal wall thickened and their uptake of glucose rose according to PET-CT. It is regretful that an invasive enteroscopy was not performed for this patient in order to get a timely treatment. The ileocecum in our case revealed no obvious abnormality at endoscopy, we therefore did not perform biopsy in the terminal ileum. It was noted however that the high concentration of lymphoid tissue in the terminal ileum was found by biopsy in this patient through colonoscopy reexamination. This offers our lesson is endoscopists should routinely observe the terminal ileum, even take biopsy specimens if necessary, which could help comprehensively evaluate the conditions.

ITCLs are aggressive hematological tumors with a poor prognosis and limited treatment strategies. The median overall survival has been demonstrated to be around 1 year.^[16]^ Low-risk international prognostic index(IPI), early diagnosis, and high-dose chemotherapy, as well as autologous stem cell therapy, can enhance prognosis.^[5,17]^ The recommended initial treatment plan for ITCL is CHOP. Probably illness recognition and timely care seeking, the patient is still with controlled disease by intensity reduction of CHOP regimen even though developed chemotherapy-related side effects. We’ll be keeping a careful eye on this patient to find out their long-term prognosis.

## 4. Conclusions

ITCL has an insidious onset, lacks specific clinical symptoms, and endoscopic, and histological findings, should arouse the attention of endoscopists. In the individual with unexplained anemia and waste, endoscopy should not be delayed. For each of polypoid lesion on white-light endoscopy, the endoscopist needs to remain cautious, because every lesion in the same patient can exhibit the independence of histopathological features. Endoscopists ought to get biopsy samples at several locations, maybe even involving the terminal ileum if necessary, which could help comprehensively evaluate the conditions and lead to better patient outcomes.

## Author contributions

**Writing – original draft:** Hanxin Bi, Junfang Bai.

**Writing – review & editing:** Limei Wang, Cong Liang, Ying Wu.
